# Away with Cosmetics

**Published:** 1876-12

**Authors:** 


### *AWAY WITH COSMETICS.*

Abandon all washes, emulsions, and lotions for the complexion. They are always either useless or dangerous. Remove the cause of sp[illegible] pimples, morphew, and other external disfigurements of an eruptive type, by removing the inward cause with a few doses of "Stafford's Iron and Sulphur Powders," which at once tone the stomach and disinfect the vital fluid. They are the finest combination of an invigorant and an antiseptic that the world has ever known.

Sold by Druggists. 1 Package, 12 Powders, $1; 6 Packages, 72 Powders, $5. Mailed Free. Hall & Ruckel, 218 Greenwich Street, N. Y.

